# Supplementary material for: Phenotypic Variation in Clinical S. aureus Isolates Did Not Affect Disinfection Efficacy Using Short-Term UV-C Radiation
Source: Microorganisms. 2023 May 18;11(5):1332. doi: 10.3390/microorganisms11051332 (PMC10223295; doi:10.3390/microorganisms11051332)
Supplement: Supplementary file 1 [file microorganisms-11-01332-s001.zip › Supplementary Figure S1.pdf]

Supplementary Figure

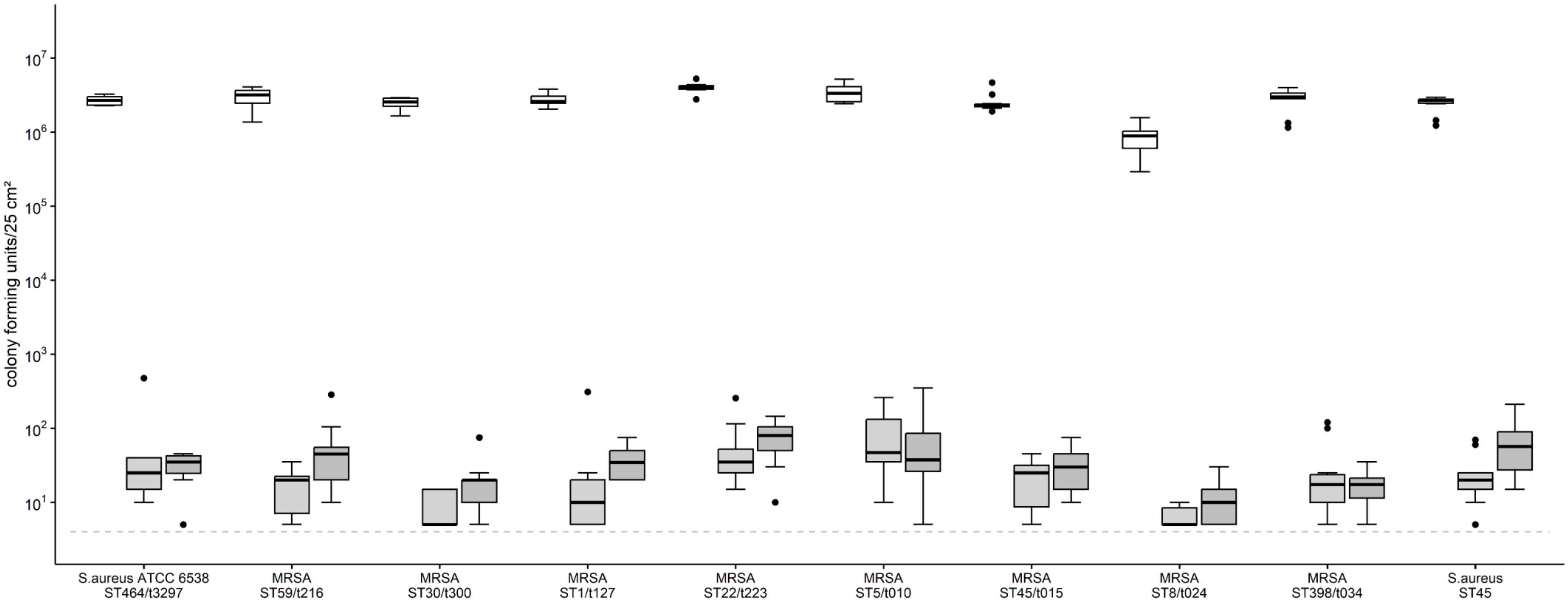

Figure S1. Bacterial contamination of surface before and after UV-C radiation with different UV-C doses. The white filled boxplots represent number of colony forming units on 25cm² ceramic tiles before UV-C disinfection. Light-grey and grey filled boxplots display the bacterial contamination after UV-C radiation with 50 and 22 mJ/cm², respectively. Each boxplot contains 10 values determined in five independent experiments. The dashed line shows the detection limit of 5 cfu/cm².
