# Supplementary material for: Phenotypic Variation in Clinical S. aureus Isolates Did Not Affect Disinfection Efficacy Using Short-Term UV-C Radiation
Source: Microorganisms. 2023 May 18;11(5):1332. doi: 10.3390/microorganisms11051332 (PMC10223295; doi:10.3390/microorganisms11051332)
Supplement: Supplementary file 1 [file microorganisms-11-01332-s001.zip › Supplementary Table S1.pdf]

Supplementary Table S1. Phenotypic and genotypic resistance in the investigated strains (only antibiotics with detected resistance mechanisms are displayed)

strain	aminoglycoside		beta-lactams			fluoroquinolones			MLS				tetracyclin	
	phenotype[a]	genotype[b]	phenotype[a]		genotype[b]	phenotype[a]		genotype[b]	phenotype[a]			genotype[b]	phenotype[a]	genotype[b]
	gentamycin		cefoxitin-screen	oxacillin		ciprofloxacin	moxifloxacin		clindamycin IR[c]	Erythromycin	cindamycin		teracyclin	
MRSA ST5/t010	≤ 0.5 (S)	aadD, bleO	positive	≥ 4 (R)	blaZ, mecA	≤ 0.5 (I)	≤ 0.25 (S)	-	negative	≤ 0.25 (S)	≤ 0.25 (S)	-	≥ 16 R	tet(M)
MRSA ST8/t024	≤ 0.5 (S)	-	positive	≥ 4 (R)	blaZ, mecA	≤ 0.5 (I)	≤ 0.25 (S)	-	negative	≤ 0.25 (S)	≤ 0.25 (S)	-	≤ 1 (S)	-
MRSA ST22/t223	≤ 0.5 (S)	-	positive	≥ 4 (R)	blaZ, mecA	≤ 0.5 (I)	≤ 0.25 (S)	-	negative	≤ 0.25 (S)	≤ 0.25 (S)	-	≥ 16 R	tet(K)
MRSA ST45/t015	4 (R)	aac(6')-aph(2'')	positive	≥ 4 (R)	blaZ, mecA	≤ 0.5 (I)	≤ 0.25 (S)	grlA p.I45M	negative	≤ 0.25 (S)	≤ 0.25 (S)	-	≤ 1 (S)	-
MRSA ST30/t300	≤ 0.5 (S)	-	positive	≥ 4 (R)	blaZ, mecA	≤ 0.5 (I)	≤ 0.25 (S)	-	positive	≥ 8 (R)	≤ 0.25 (R)[c]	erm(C)	≥ 16 R R	tet(K)
MRSA ST59/t216	≤ 0.5 (S)	-	positive	≥ 4 (R)	blaZ, mecA	≤ 0.5 (I)	≤ 0.25 (S)	-	negative	≤ 0.25 (S)	≤ 0.25 (S)	-	≤ 1 (S)	-
MRSA ST1/t127	≤ 0.5 (S)	aph(3')-III, ant(6)-Ia	positive	≥ 4 (R)	blaZ, mecA	≤ 0.5 (I)	≤ 0.25 (S)	-	positive	≥ 8 (R)	≤ 0.25 (R)[c]	erm(C)	≥ 16 R	tet(K)
MRSA ST398/t034	≤ 0.5 (S)	ant(9)-Ia, str	positive	≥ 4 (R)	blaZ, mecA	1 (I)	≤ 0.25 (S)	-	negative	≥ 8 (R)	≥ 8 (R)	erm(A)	≥ 16 R	tet(M)
MSSA ST45	≤ 0.5 (S)	-	negative	≤ 0.25 (S)	-	≤ 0.5 (I)	≤ 0.25 (S)	-	negative	≤ 0.25 (S)	≤ 0.25 (S)	-	≤ 1 (S)	-
MSSA ATCC 6538	≤ 0.5 (S)	-	negative	≤ 0.25 (S)	-	≤ 0.5 (I)	≤ 0.25 (S)	grlA p.I45M	negative	≤ 0.25 (S)	≤ 0.25 (S)	-	≤ 1 (S)	-

[a]Values represent minimal inhibitory concentrations (mg/L) as detected by VITEK AST as well as the S-I-R classification (in parenthesis) according to EUCAST; Results of screening reactions are presented as positive or negative
[b]Genes or mutations (fluoroquinolones) detected in the genome of sequenced isolates are indicated if present
[c]Inducible resistance (IR) against clindamycin resulting in the classification as resistant (R) despite MIC values ≤ 0.25 mg/L
